# *MBD4* frameshift mutation caused by DNA mismatch repair deficiency enhances cytotoxicity by trifluridine, an active antitumor agent of TAS-102, in colorectal cancer cells

**DOI:** 10.18632/oncotarget.22484

**Published:** 2017-11-15

**Authors:** Satoshi Suzuki, Moriya Iwaizumi, Hidetaka Yamada, Tomohiro Sugiyama, Yasushi Hamaya, Takahisa Furuta, Shigeru Kanaoka, Haruhiko Sugimura, Hiroaki Miyajima, Satoshi Osawa, John M. Carethers, Ken Sugimoto

**Affiliations:** ^1^ First Department of Medicine, Hamamatsu University School of Medicine, Hamamatsu, Japan; ^2^ Department of Tumor Pathology, Hamamatsu University School of Medicine, Hamamatsu, Japan; ^3^ Center for Clinical Research, Hamamatsu University School of Medicine, Hamamatsu, Japan; ^4^ Department of Gastroenterology, Hamamatsu Medical Center, Hamamatsu, Japan; ^5^ Department of Endoscopic and Photodynamic Medicine, Hamamatsu University School of Medicine, Hamamatsu, Japan; ^6^ Division of Gastroenterology, Department of Internal Medicine, University of Michigan, Ann Arbor, Michigan, USA; ^7^ International Mass Imaging Center, Hamamatsu University School of Medicine, Hamamatsu, Japan

**Keywords:** colorectal cancer, trifluridine, MBD4, microsatellite instability, frameshift mutation

## Abstract

**Backgrounds:**

Trifluridine is an active antitumor component of TAS-102 that resembles 5-fluorouracil. Although patients with advanced colorectal cancer (CRC) exhibiting a mismatch repair (MMR) deficiency reportedly do not benefit from 5-fluorouracil-based chemotherapy and we previously reported that truncated methyl-CpG binding domain protein 4 (MBD4) enhances 5-fluorouracil cytotoxicity in MMR-deficient CRC cells, little is known regarding the effect of MMR deficiency on trifluridine cytotoxicity in CRC.

**Aim:**

We investigated whether trifluridine induces cytotoxicity in a DNA MMR-dependent manner and evaluated how truncated MBD4 alters trifluridine cytotoxicity.

**Methods:**

We utilized the human CRC cell lines HCT116 (hMLH1-deficient cells) and HCT116+ch3 (hMLH1-restored cells) and compared their sensitivities to trifluridine. And we established 5-fluorouracil-refractory hMLH1-deficient cells and analyzed trifluridine cytotoxicity. Finally, we established truncated MBD4 overexpressed CRC cell lines, and compared trifluridine sensitivity.

**Results**

The sensitivities of HCT116 and HCT116+ch3 to trifluridine were comparable. 5-Fluorouracil-refractory hMLH1-deficient cells treated with trifluridine showed an equal or greater sensitivity than non-5-fluorouracil-refractory cells. Moreover, MBD4tru cells were more sensitive than the control cells to trifluridine.**Conclusions:** Trifluridine induces cytotoxicity independently of the DNA MMR status as well as under 5-fluorouracil-refractory conditions, and the *MBD4* frameshift mutation enhances trifluridine cytotoxicity.

## INTRODUCTION

Fluoropyrimidine-based chemotherapy is the cornerstone of chemotherapy in patients with advanced or metastatic colorectal cancer (CRC). Nowadays, standard fluoropyrimidine- based treatment options for metastatic CRC are varied by combination with other cytotoxic agents (such as oxaliplatin or irinotecan), biological targeted agents (such as bevacizumab and aflibercept), or anti EGFR antibodies, these treatments benefit patients with CRC that are refractory to first-line chemotherapy as well as those who are chemotherapy-naive. [1] In addition to the disease stage, the tumor’s DNA mismatch repair (MMR) status appears to predict the response to the fluoropyrimidine 5-fluorouracil (5-FU). [2] Several reports have suggested that 5-FU is not effective against colon cancer with MMR deficiency. [3–7] In other words, patients with sporadic microsatellite unstable (MSI) cancers (mainly hypermethylation of the DNA MMR gene hMLH1 [8]) or Lynch syndrome (a germline mutation in a DNA MMR gene [9]) are unlikely to benefit from 5-FU-based chemotherapy, compared with patients with microsatellite stable (DNA MMR- proficient) tumors. Experimentally, it has been estimated that as much as 10% of cellular 5-FU is incorporated into DNA and is recognized by hMutSα, a heterodimer of the DNA MMR proteins hMSH2 and hMSH6, or hMutSβ, an hMSH2-hMSH3 heterodimer, [10, 11] and the incorporation of 5-FU into DNA induces cytotoxicity in a DNA MMR-dependent manner. [12, 13] These observations remind us that the DNA MMR function or microsatellite instability status of tumors might change the cytotoxicity of other kinds of fluoropyrimidine analogues.

TAS-102 is a novel oral anti-folate drug containing the thymidine-based nucleic acid analogue trifluridine (FTD) and tipiracil hydrochloride, which is an inhibitor of the FTD degradation enzyme thymidine phosphorylase (TPI). [14] A double-blinded, randomized, placebo-controlled, phase 2 trial for patients with metastatic CRC refractory to 5-FU showed that the median overall survival period was 9.0 months in the TAS-102 group and 6.6 months in the placebo group. [15] TAS-102 was further evaluated using a double-blinded, placebo- controlled, phase 3 trial involving 800 patients with metastatic CRC, and the medial overall survival period was 7.1 months with TAS-102, whereas it was 5.3 months with a placebo, [16] suggesting that TAS-102 exerted a clinical activity in a large population of Japanese and Western patients with metastatic CRC refractory to 5-FU. FTD is an active antitumor agent of TAS-102, and its chemical structure is similar to that of the clinically active 5-FU analogue, 2'-deoxy-5-fluorouridine (FdUrd), in that FTD is a thymidine analogue and FdUrd is a 5-FU uridine analogue. [17].

After FTD is phosphorylated by intercellular thymidine kinase and forms FTD-monophosphate (F3dTMP), which inhibits thymidylate synthase (TS), and then forms FTD-triphosphate (F3dTTP), F3dTTP is incorporated into DNA as 5-FU. However, the magnitude of FTD incorporation into DNA is much higher than that of 5-FU. [18] These observations indicate that the incorporation of FTD into DNA causes cytotoxicity, and the DNA MMR status affects the chemosensitivity of CRC cells to FTD, similar to its effect on 5-FU sensitivity.

Although it is clinically reported that patients with tumors that lost DNA mismatch repair (MMR) system does not have a benefit from 5-FU based adjuvant chemotherapy, none is reported if MMR deficiency inhibits FTD cytotoxicity. Here, we utilized human hMLH1-deficient and hMLH1-restored CRC cells and established 5-FU-refractory hMLH1-deficient cells by continuous 5-FU exposure for 10-12 months. We then examined FTD cytotoxicity in cells with different DNA MMR statuses.

In addition, we examined FTD cytotoxicity caused by protein modification arising from MMR deficiency. We focused on the uracil DNA glycosylase methyl-CpG binding domain protein 4 (MBD4), which not only excises 5-FU from DNA but also leads to a frameshift mutation caused by MSI. MBD4 is a methyl-CpG binding DNA glycosylase involved in the repair of mismatches arising from the deamination of methyl-cytosine (C) in mammalian cells, [19] and catalyzes the removal of thymine (T) and uracil (U) paired with guanine within CpG sites. [20] MBD4 is frequently mutated in human colorectal carcinoma cells, most commonly by frameshifts in the A10 repeat at position 301–310 (31–33), thereby disrupting downstream sequences including the glycosylase and domain of MLH1 interaction. [21–24] Because we previously reported that truncated MBD4 enhances 5-FU cytotoxicity in MMR-deficient CRC cells, [25] the question of whether truncated MBD4 also induces FTD cytotoxicity was raised. Here, we investigated how truncated MBD4 alters FTD cytotoxicity using our established truncated MBD4-overexpressed MMR-deficient cell line.

## RESULTS

### Colorectal cancer cells with DNA mismatch repair deficiency are sensitive to FTD

**Figure 1:**
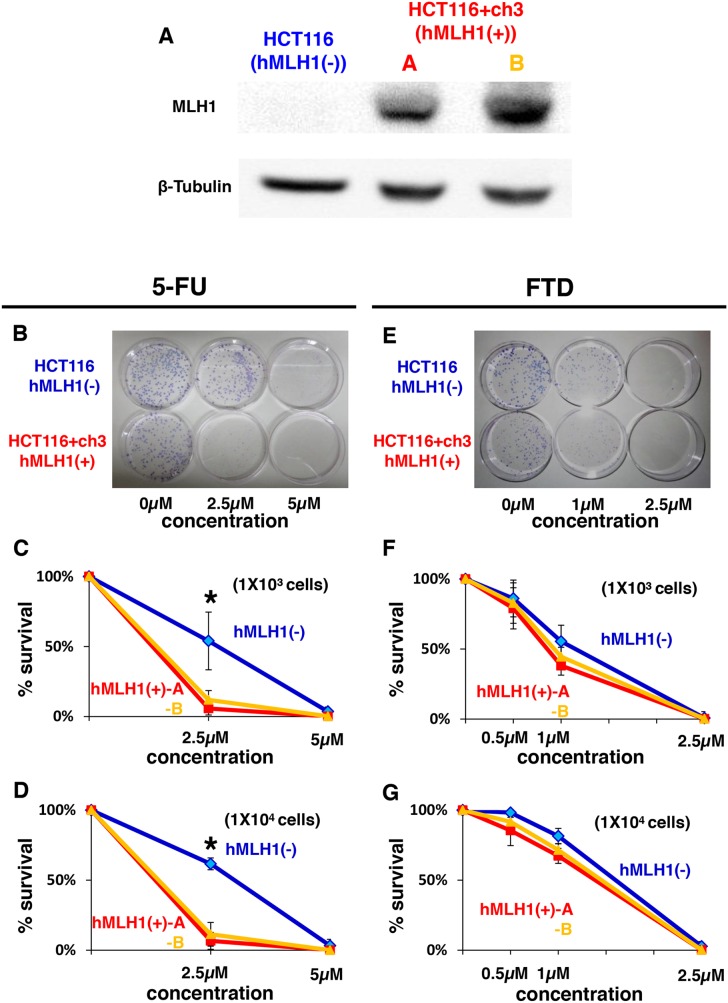
Sensitivity of MMR-deficient cells to FTD treatment is the same as that of MMR-proficient cells despite MMR-deficient cells being resistant to 5-FU **(A)** Results of western blot. Left lane: HCT116 (hMLH1(−)), middle lane: HCT116+ch3-A (hMLH1(+)), right lane: HCT116+ch3-B (hMLH1(+)). **(B-G)** Clonogenic assay of HCT116, HCT116+ch3-A, and HCT116+ch3-B in response to 5-FU. A total of 1 × 10^3^ cells per 100-mm dish (B, C) or 1 × 10^4^ cells per 100-mm dish (D) were plated in media containing 0, 2.5, or 5 μM of 5-FU and were allowed to form colonies over 10 days. (E, F, G) Same procedure as before with FTD (0, 0.5, 1 or 2.5 μM). Each experiment was performed in triplicate, and the experiment was replicated three independent times. Data were expressed as the mean±SE. ^*^ *P*< 0.05.

First, we performed a clonogenic assay with different initial cell numbers (1 × 10^3^ cells/100-mm dish and 1 × 10^4^ cells/100-mm dish) to confirm the results of previous reports indicating that DNA MMR-deficient cells are resistant to 5-FU [11–13] using the HCT116 (hMLH1(−)) and HCT116+ch3 (hMLH1(+)) cell lines. (Figure 1A) As others have previously demonstrated, the area of colonies of hMLH1(−) cells was larger than the area of colonies of hMLH1(+) cells when 1 × 10^3^ cells were treated with 2.5 μM of 5-FU (*P* < 0.05) (Figure 1B and 1C), and these results were confirmed by treating 1 × 10^4^ cells with 5-FU (Figure 1D). When we treated 1 × 10^3^ cells with FTD, the area of colonies of hMLH1(−) cells was the same as the area of colonies of hMLH1(+) cells (Figure 1E and 1F), and these results were confirmed using 1 × 10^4^ cells (Figure 1G). These results indicate that FTD induces cytotoxicity in DNA MMR-deficient cells to the same extent as that in MMR-proficient cells, even though DNA MMR-deficient cells are resistant to 5-FU.

### FTD effectively induces cytotoxicity in colorectal cancer cells refractory to 5-FU

**Figure 2:**
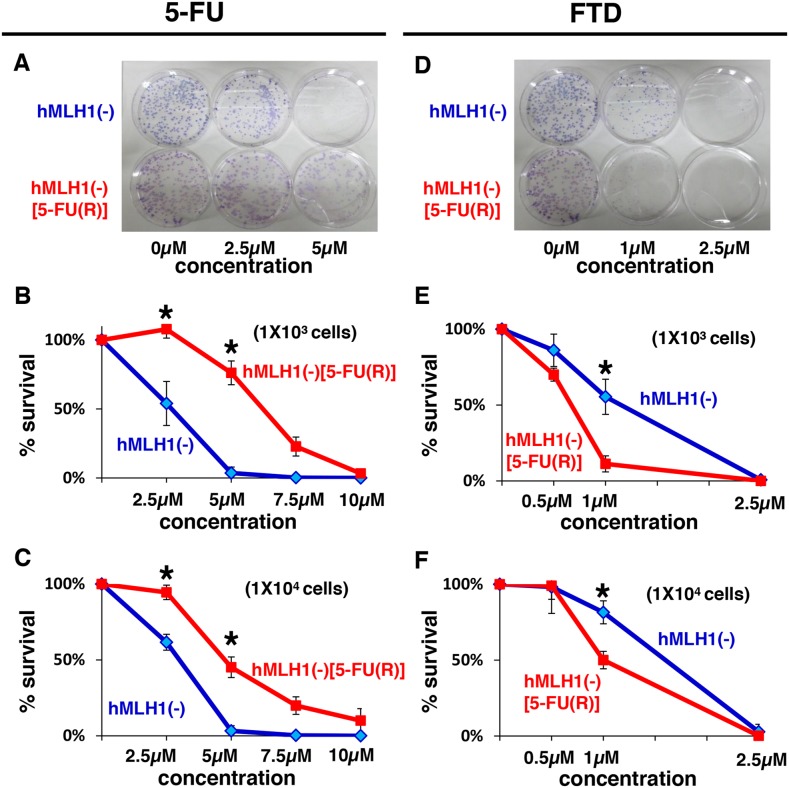
MMR-deficient cells that are refractory to 5-FU are sensitive to FTD Clonogenic assay of HCT116 (hMLH1(−)) and 5-FU resistant HCT116 (hMLH1(−) [5-FU(R)]). A total of 1 × 10^3^ cells per 100-mm dish **(A, B)** or 1 × 10^4^ cells per 100-mm dish **(C)** were plated in media containing 0, 2.5, or 5 μM of 5-FU and were allowed to form colonies over 10 days. Same procedure as before with FTD (0, 0.5, 1 or 2.5 μM) **(D, E, F)**. Each experiment was performed in triplicate, and the experiment was replicated three independent times. Data were expressed as the mean±SE. ^*^P < 0.05.

Since DNA MMR-deficient cells were shown to be sensitive to FTD despite being resistant to 5-FU, we next examined whether 5-FU-refractory cells were also sensitive to FTD, since TAS-102, which consists of FTD and tipiracil hydrochloride (a thymidine phosphorylase inhibitor) had been clinically shown to be effective for patients with metastatic CRC whose diseases are refractory to fluorouracil. [15, 16] To answer the question, we continuously exposed HCT116 cells to 5-FU for 10-12 months and then selected viable colonies, which were super-resistant to 5-FU because they lacked DNA MMR-function and were refractory to 5-FU after having been exposed to 5-FU for a long time period. We then compared the cytotoxicity after 5-FU treatment between hMLH1(−) cells and 5-FU- refractory-hMLH1(−) (hMLH1(−) [5-FU(R)]) cells using a clonogenic assay, and the area of colonies of hMLH1(−)[5-FU(R)] cells was larger than the area of colonies of hMLH1(−) cells (*P* < 0.05)(Figure 2A, 2B, 2C), confirming that we had successfully established hMLH1- deficient 5-FU-refractory cells. We next used these hMLH1(−) [5-FU(R)] cells and compared FTD cytotoxicity with that of hMLH1(−) cells using a clonogenic assay. Interestingly, the area of colonies of hMLH1(−) [5-FU(R)] was smaller than that of hMLH1(−) cells when 1× 10^3^ cells were treated with 1μM of FTD (*P* < 0.05) (Figure 2D and 2E); these results were then confirmed by treating 1 × 10^4^ cells with FTD (Figure 2F). Not only are these results analogous to clinical evidence of the effectiveness of TAS-102 for patients with metastatic CRC that is refractory to 5-FU-based chemotherapy, but they also indicate that TAS-102 may be more effective for patients with tumors that are refractory to 5-FU than for patients with 5-FU-naive tumors.

### *MBD4* frameshift mutation enhances FTD sensitivity through G2/M arrest in colorectal cancer cells

**Figure 3:**
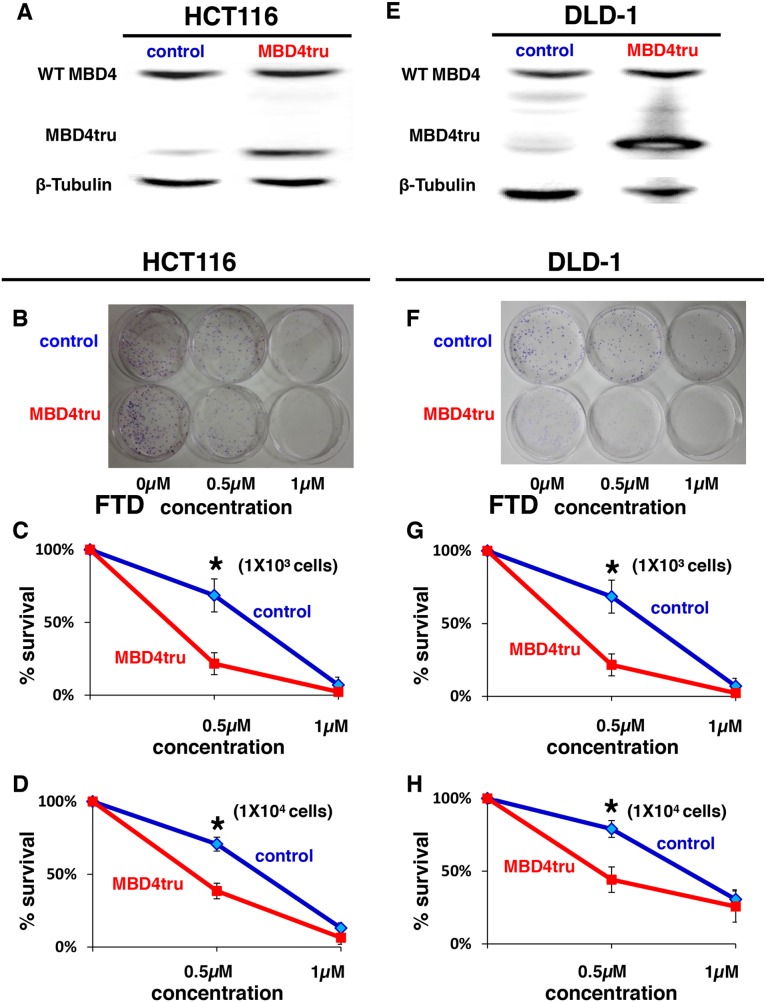
*MBD4* frameshift mutation caused by MSI enhances FTD cytotoxicity **(A)** Results of western blot. Left lane: HCT116 transfected empty pcDNA 3.1(+) plasmid vector (HCT116-control), right lane: HCT116 transfected pcDNA 3.1(+) plasmid vector encoding a truncated MBD4 (HCT116-MBD4tru). **(B-D)** Clonogenic assay of HCT116-control and HCT116-MBD4tru in response to FTD. A total of 1 × 10^3^ cells per 100-mm dish (B, C) or 1 × 10^4^ cells per 100-mm dish (D) were plated in media containing 0, 0.5, or 1 μM of FTD and were allowed to form colonies over 10 days. **(E)** Results of western blot. Left lane: DLD-1 transfected empty pcDNA 3.1(+) plasmid vector (DLD-1 control), right lane: DLD-1 transfected pcDNA 3.1(+) plasmid vector encoding a truncated MBD4 (DLD-1-MBD4tru). **(F-H)** Clonogenic assay of DLD-1-control and DLD-1-MBD4tru in response to FTD. A total of 1 × 10^3^ cells per 100-mm dish (F, G) or 1 × 10^4^ cells per 100-mm dish (H) were plated in media containing 0, 0.5, or 1 μM of FTD and were allowed to form colonies over 10 days. Each experiment was performed in triplicate, and the experiment was replicated three independent times. Data were expressed as the mean±SE. ^*^P < 0.05.

Based on the cell growth data indicating that the MSI status contributes to the enhancement of sensitivity to FTD in DNA MMR-deficient cells, we focused on uracil DNA glycosylases (UDGs) that excise FdUrd from DNA. Among the 4 known UDGs that excise FdUrd from DNA, i.e., methyl-CpG binding domain protein 4 (MBD4), [26] thymine DNA glycosylase (TDG), [27] singlestrand-selective monofunctional uracil-DNA glycosylase 1(Smug1), [28] and uracil-DNA glycosylase (UNG), [29] only MBD4 is known to lead to an MSI-induced frameshift mutation. [2] Therefore, we established HCT116 cells that stably express truncated MBD4 (MBD4tru) using a frameshift mutation of *MBD4* by A10 to A9 deletion at codons 310-313, which is clinically seen in DNA MMR-deficient CRC and that lacks a glycosylase domain but in which the methyl CpG bindingdomain is conserved (Figure 3A). [23] Interestingly, we observed in a clonogenic assay that the area of colonies in MBD4tru cells was smaller than the area of control cells when treated with 0.5 μM or 1 μM of FTD in 1 × 10^3^ cells (*P* < 0.05) (Figure 3B, 3C) and confirmed the result in 1 × 10^4^ cells (Figure 3D).

To investigate change of cytotoxicity from truncated MBD4 in other MMR status, we established DLD-1 cells that stably express truncated MBD4 (Figure 3E) and conducted clonogenic assay. As with HCT116, we observed in a clonogenic assay that the area of colonies in MBD4tru cells was smaller than the area of control cells when treated with 0.5 μM or 1 μM of FTD in 1 × 10^3^ cells (P < 0.05) (Figure 3F, 3G) and confirmed the result in 1 × 10^4^ cells (Figure 3H).

**Figure 4:**
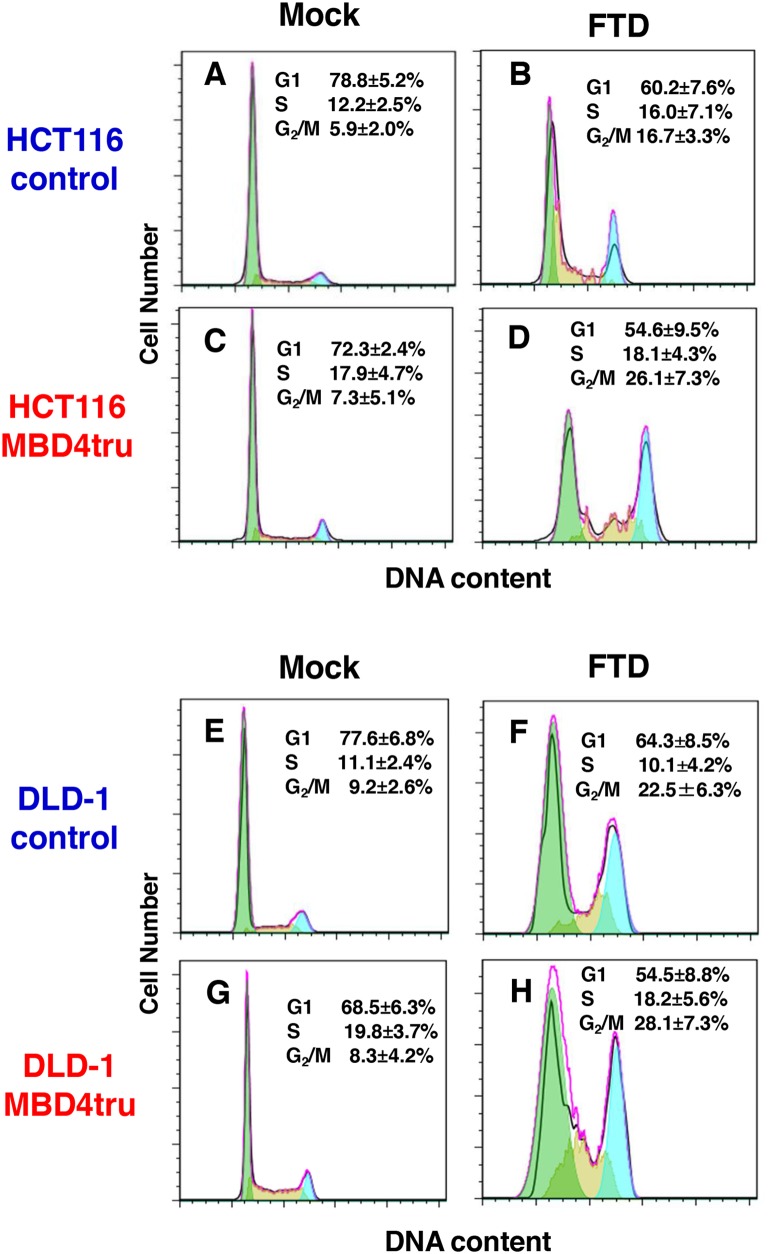
*MBD4* frameshift mutation caused by MSI enhances FTD cytotoxicity through G_2_/M arrest in colorectal cancer cells Analysis of cell cycle using fluorescence-activated cell sorting analysis (FACS) with propidium iodide staining. We incubated cell lines for 3 days under FTD treatment (1 μM). **(A)** HCT116-control (Mock), **(B)** HCT116-control (FTD treated), **(C)** HCT116-MBD4tru (Mock), **(D)** HCT116-MBD4tru (FTD treated), **(E)** DLD-1-control (Mock), **(F)** DLD-1-control (FTD treated), **(G)** DLD-1-MBD4tru (Mock), **(H)** DLD-1-MBD4tru (FTD treated). Each experiment was performed in triplicate, and the experiment was replicated three independent times. Data were expressed as the mean±SD.

To further analyze how FTD induces cytotoxicity by truncated MBD4 expression, we performed fluorescence-activated cell sorting analysis (FACS) with propidium iodide staining of cells that had asynchronously grown for 3 days under FTD treatment (1 μM). When HCT116-control and HCT116-MBD4tru were treated with FTD, the G2/M population was larger than that with no FTD treatment (Figure 4A, 4B, 4C and 4D). Furthermore, the rate of G2/M increase was slightly higher in HCT116-MBD4tru than in HCT116-control. When DLD-1-control and DLD-1-MBD4tru were treated with FTD, the G2/M population was larger than that with no FTD treatment (Figure 4E, 4F, 4G and 4H). The rate of G2/M increase was slightly higher in DLD-1-MBD4tru than in DLD-1-control. Our observations suggest that the expression of truncated MBD4 produced by an *MBD4* frameshift mutation in the presence of DNA MMR deficiency enhanced FTD cytotoxicity by inducing G2/M arrest.

## DISCUSSION

Clinical evidence has suggested that patients with advanced colorectal cancer whose tumors are refractory to 5-FU might benefit from treatment with TAS-102, which contains the 5-FU analogue FTD and TPI. [15, 16] Although both FTD and the 5-FU metabolite FdUrd are incorporated into DNA, resulting in cytotoxicity, and their chemical structures are similar in that both of them are thymidine/uracil derivatives, [6, 17, 18] there is no data regarding the DNA MMR effect in the cytotoxicity of FTD, despite the fact that the cytotoxicity of 5-FU reportedly depends on the DNA MMR status of cells.

Our study demonstrated that (a) cells with DNA MMR deficiency are sensitive to FTD to the same extent as cells with DNA MMR proficiency despite DNA MMR-deficient cells being more resistant to 5-FU than DNA MMR-proficient cells, (b) cells that are resistant to 5-FU as a result of DNA MMR deficiency and continuous 5-FU exposure are sensitive to FTD, and (c) and that DNA MMR-deficient cells with truncated MBD4 expression produced by a *MBD4* frameshift mutation exhibit enhanced FTD cytotoxicity as a result of G2/M arrest. This is the first study to demonstrate the contribution of *MBD4* frameshift mutation resulting from DNA MMR deficiency to the enhancement of FTD cytotoxicity.

Utilizing CRC cells with hMLH1 deficiency, which are representative of the majority of MSI-positive sporadic CRCs, our data confirmed some previous experimental reports that DNA MMR-deficient CRC cells are resistant to 5-FU [10–13] and support some clinical evidence that patients with stage II CRC with tumors that lost DNA MMR function do not derive a benefit from 5-FU-based chemotherapy. [6, 7] Notably, the clinical practice guidelines of the National Comprehensive Cancer Network (NCCN) state that tumor immunohistochemistry for DNA MMR protein or tumor MSI testing should be performed for all patients with stage II diseases when considering the use of 5-FU adjuvant therapy for patients with stage II CRC. In other words, effective adjuvant therapeutic options should be developed for patients with stage II CRC whose tumors are MSI-positive. Our data showing a similar FTD cytotoxicity for both hMLH1-deficient and hMLH1-restored cells suggest that TAS-102 is a candidate for adjuvant therapy for stage II patients with MSI-positive CRC.

FTD resembles the 5-FU metabolite FdUrd in its chemical structure, but their roles in cytotoxicity seem to differ somewhat in terms of (I) the magnitude of their misincorporation into DNA resulting in cytotoxicity, and (II) the inhibitory power of thymidylate synthase (TS). Both FTD and FdUrd inhibit TS in their monophosphate form (F3dTMP from FTD and FdUMP from FdUrd, respectively) produced by thymidine kinase (TK), [30] and the monophosphate forms are changed to their triphosphate form through their diphosphate form (FTD to F3dTTP through FTD-diphosphate [F3dTDP] and FdUrd to FdUrd-triphosphate [FdUTP] through FdUrd-diphosphate [FdUDP], respectively). The triphosphate forms are then incorporated into the DNA of the cells and induce cytotoxicity. [17] Sakamoto et al. showed that a salvage pathway from the triphosphate form to the monophosphate form is much more active in FuUrd metabolism than in FTD metabolism because FdUTP is a substrate for deoxyUTPase (DUT), but F3dTTP is not. [17] Therefore, the concentration of monophosphate forms is lower for FTD than for FdUrd, whereas the concentration of triphosphate forms is higher for FTD than for FdUrd, leading to a higher magnitude of the misincorporation of FTD into DNA than that of FdUrd for cytotoxicity. [17, 18] In this regards, it is possible that excessive FTD incorporated into DNA may be recognized by DNA repair systems (i.e., base excision repair [BER]) that excise FTD from DNA other than the DNA MMR system and that maintain cytotoxicity even under DNA MMR-deficient condition.

Our additional data utilizing 5-FU-refractory DNA MMR-deficient cells established by continuous 5-FU exposure supported clinical evidence that TAS-102 is effective for patients with metastatic CRC whose diseases are refractory to fluorouracil. [15, 16] Nakamura et al. also established 5-FU-resistant gastric cancer cell lines using continuous 5-FU exposure and showed that 5-FU-resistant cells had a 1.9-fold to 3.5-fold higher TS mRNA expression level. [31] Niedzwieckia et al. reported that tumors with high TS expression were more likely to demonstrate MMR deficiency. [32] The 5-FU-resistant HCT116 cell line established in our study might also have a high TS expression level, and our data may support previous data reported by other groups showing that FTD induces cytotoxicity independently of the TS expression level, [17] suggesting that the DNA damage response (DDR) after the misincorporation of FTD into DNA is the main mechanism of action in FTD cytotoxicity, rather than TS inhibition.

What is the key player in enhancing FTD cytotoxicity after its misincorporation into DNA? Because FTD structurally resembles FdUrd, it is natural to consider the contribution of known human DNA glycosylases that excise FdUrd in DNA, such as MBD4 or TDG. The contribution of TDG or MBD4 was apparently negligible, as Suzuki et al. demonstrated that the knockdown of MBD4 or TDG does not change FTD sensitivity in HeLa cells. [18] However, MBD4 has an A10 repeat coding sequence at codons 310-313, and some groups have clinically detected a frameshift mutation arising from an A10 to A9 deletion that produces a truncated MBD4 protein lacking both the glycosylase domain and the hMLH1 binding domain in MSI-positive colorectal cancer. [22, 23, 33, 34] In an experiment, Bader et al. demonstrated that the expression of truncated MBD4 in MSI CRC cell lines increased the mutation frequency during cancer progression, [33] and Abdel-Rahman et al. demonstrated that human CRC cell lines with truncated MBD4 expression are sensitive to cisplatin, which damages the DNA by causing intrastrand G-G adducts, but are resistant to etoposide which inhibits topoisomerase II. [19] Notably, among the four known UDGs that excise FdUrd from DNA (MBD4, TDG, Smug1, and UNG), only *MBD4* is known to have high frameshift mutation rates in MSI tumors. [2] We had reported that MBD4tru enhances 5-FU cytotoxicity in MSI CRC cells. [25] By focusing on the mechanism for FTD cytotoxicity and the widespread clinical and biological effects of truncated MBD4 in CRC, we were able to establish truncated MBD4-expressing, MSI-positive cells and show the enhancement of FTD cytotoxicity through G2/M arrest.

In conclusion, we demonstrated that FTD is effective to 5-FU-refractory colon cancer cell independently of the DNA MMR status, and that *MBD4* frameshift mutation caused by DNA MMR deficiency enhance FTD sensitivity through G2/M arrest. Our data indicates that TAS-102-based therapy is effective not only for patients with 5-FU-refractory CRC, but also for stage II CRC patients with MSI-H tumors who do not benefit from 5-FU-based adjuvant therapy or 5-FU-treatment-naive patients with MSI-H/*MBD4* frameshift mutant CRC. Further clinical studies are needed.

## MATERIALS AND METHODS

### Cell lines

The human colon cancer cell line HCT116 (*hMLH1−/−*) and DLD-1 (*hMSH6−/−*) were obtained from the American Type Culture Collection (Rockville, MD, USA), and the HCT116+ch3 cell line (HCT116+ch3-A) was an hMLH1-restored cell line that was a present from Dr. John M. Carethers (University of Michigan, MI, USA). [35] Another HCT116+ch3 cell line (HCT116+ch3-B) was a present from Dr. Hiromichi Henmi (Toho University, JAPAN). The HCT116 cells were maintained in Iscove’s modified Dulbecco’s medium (IMDM; Invitrogen, Carlsbad, CA) with 10% fetal bovine serum and penicillin (100U/mL)/streptomycin (100μg/mL) (Invitrogen) as a supplement at 37°C in a 5% CO2/95% air incubator. The DLD-1 cells were maintained in Roswell Park Memorial Institute medium 1680(RPMI1680; Invitrogen) with 10% fetal bovine serum and penicillin (100U/mL)/streptomycin (100μg/mL) (Invitrogen) as a supplement at 37°C in a 5% CO2/95% air incubator. The HCT116+ch3 cells were maintained in IMDM (Invitrogen) with 10% fetal bovine serum and penicillin (100U/mL)/streptomycin (100μg/mL) (Invitrogen) and 400μg/mL of G418 (GIBCO BRL, Gaithersburg, MD, USA).

### Reagents

5-FU was obtained from Sigma Chemical Co. (St. Louis, MO, USA) and was dissolved in 10% dimethylsulfoxide (DMSO) at a stock concentration of 1mM and maintained at 4°C. FTD was also obtained from Sigma Chemical Co. (St. Louis, MO, USA) and was dissolved in 10% DMSO at a stock concentration of 10mM and maintained at −20°C.

### Establishment of hMLH1-deficient cells refractory to 5-FU

To establish hMLH1-deficient cells that were refractory to 5-FU (hMLH1(−) [5-FU[R]), we modified Takahashi’s procedure for 5-FU exposure. [36] Briefly, HCT116 was exposed to an initial 5-FU concentration of 0.5 μM in IMDM plus 10% FBS. The drug concentration was then increased 1.2-2 times at each step of resistance, from 0.5μM up to 7.5μM. The cells were cultured for at least four weeks at each step; the medium was exchanged every three days.

### Transfection

To isolate a stable truncated MBD4-overexpressed MMR-deficient clone, HCT116 and DLD-1 cells were transfected with a pcDNA 3.1(+) plasmid vector (Invitrogen) encoding a truncated *MBD4* arising from a frameshift mutation of *MBD4* by an A10 to A9 deletion at codons 310- 313 and that lacked a glycosylase domain using the Nucleofector Kit V (for HCT116) and Kit L (for DLD-1) (Lonza, Germany). Selection was performed using 400μg/mL of G418. After selection, the colonies were pooled and cultured for subsequent analysis.

By confirming truncated MBD4 expression using western blotting, we established truncated MBD4 overexpressed HCT116 (HCT116-MBD4tru) and DLD-1 (DLD-1-MBD4tru) cell lines. As a negative control, HCT116 and DLD-1 were transfected with a pcDNA3.1 (+) parental vector, selected by 400 μg/mL of G418, and established an HCT116 cell line transfected with the parental vector (HCT116-control) and a DLD-1 cell line transfected with the parental vector (DLD-1-control).

### Protein extraction and western blotting

Cultured cells were solubilized in lysis buffer (10 mM Tris-HCl [pH7.2], 150 mM NaCl, 0.5% deoxycholic acid sodium salt, 0.1% Nonidet P-40, 5 mM EDTA, and proteinase inhibitor cocktail (Sigma) on ice and then centrifuged (14,000 r.p.m) for 15 min at 4°C. The supernatant was mixed with 4 × protein sample buffer (4 × NuPAGE® LDS Sample Buffer [Life Technologies, NY, USA], 3% 2-mercaptoethanol) and heated for 10 min at 98°C. The proteins were then separated by electrophoresis on 4%-12% NuPAGE® Bis-Tris Mini Gels (Life Technologies, CA USA) and transferred to Protoran− Nitrocellulose membranes (GE Healthcare Bio-Sciences, CA, USA) in a transfer apparatus (Life Technologies). The membranes were blocked with 5% skim milk and 0.1% Tween in Tris-buffered saline (TBS). Immunodetection was done utilizing the primary antibodies; MBD4 (Sigma), β-tubline (GE Healthcare Bio-Sciences), and horseradish peroxidase (HRP) linked F(ab’)2 secondary rabbit or mouse antibodies (Santa Cruz Biotechnology, CA, USA). The signals were detected using an LAS-4000 luminescent image analyzer (GE Healthcare Bio-Sciences) utilizing a chemiluminescent solution.

### Clonogenic assay

Exponentially growing cells were trypsinized and washed twice with PBS. The cells were then plated on a 100mm Tissue Culture Dish (Becton Dickinson Labware, NJ) in IMDM (for HCT116) and RPMI1680 (for DLD-1) supplemented with 10% FBS and containing various concentrations of 5-FU or FTD, then incubated at 37°C and 5% CO2. After 10 days of growth, the culture plates were washed with PBS, fixed with methanol for 15 minutes, and then rewashed with PBS. The colonies were stained with 3% Giemsa (Sigma, St Louis, MO) for 15 minutes and rinsed with water. To quantify colony formation, we used the free distribution software ImageJ (National Institutes of Health, Bethesda, MD, USA), as previously described. [37] The relative surviving fraction for each cell line was expressed as the ratio of the plating efficiency in treated cultures to that observed in the controls.

### Fluorescence-activated cell sorting (FACS) analysis

We incubated four cell lines (HCT116-control, HCT116-MBD4tru, DLD-1-control and DLD-1-MBD4tru) for 3 days under FTD treatment (1μM). For the FACS analysis, the cells were washed in phosphate-buffered saline (PBS), incubated in PBS for 10 min, and then trypsinized and fixed in 80% ethanol. The cells were washed again, resuspended in 50 μg/mL of propidium iodide, and analyzed using a Beckman Coulter Epics XL (Beckman Coulter, CA, USA). The cell cycle analysis was performed using FlowJo software (FlowJo LLC, Ashland, Oregon, USA).

### Statistical analysis

Comparisons were made using the Student *t*-test. P values less than 0.05 were considered statistically significant. Statistical analyses were performed using StatView 5.0 software (SAS Institute, Cary, USA).

### Ethics

The design of this study, including the human genomics and recombinant DNA research, was approved by the Institutional Review Board of Hamamatsu University School of Medicine.

## Abbreviations

5-FU: 5-fluorouracil
FTD: trifluridine
TPI: thymidine phosphorylase
FdUrd: 2'-deoxy-5-fluorouridine
F3dTMP: FTD-monophosphate
F3dTDP: FTD-diphosphate
F3dTTP: FTD-triphosphate
MBD4: methyl-CpG binding domain protein 4
TDG: thymine DNA glycosylase
Smug1: singlestrand-selective monofunctional uracil-DNA glycosylase 1
UNG: uracil-DNA glycosylase
MSI-H: microsatellite instability-high
CRC: colorectal cancer
MMR: mismatch repair
FdUMP: 5-fluorodeoxyuridine monophosphate
FdUDP: 5-fluorodeoxyuridine diphosphate
FdUTP: 5-fluorodeoxyuridine triphosphate
DUT: deoxyUTPase
FBS: fetal bovine serum
